# Frailty, Fitness, and Quality of Life Outcomes of a Healthy and Productive Aging Program (GrandMove) for Older Adults With Frailty or Prefrailty: Cluster Randomized Controlled Trial

**DOI:** 10.2196/65636

**Published:** 2025-05-14

**Authors:** Jennifer Yee Man Tang, Hao Luo, Michael Tse, Joseph Kwan, Angela Yee Man Leung, Teresa Bik-kwan Tsien Wong, Terry Yat Sang Lum, Gloria Hoi Yan Wong

**Affiliations:** 1 Department of Educational Psychology, The Chinese University of Hong Kong, Shatin, China (Hong Kong); 2 School of Public Health Sciences, University of Waterloo, Waterloo, ON, Canada; 3 Department of Social Work and Social Administration, The University of Hong Kong, Hong Kong, Pok Fu Lam, China (Hong Kong); 4 Centre for Sports and Exercise, The University of Hong Kong, Hong Kong, Pok Fu Lam, China (Hong Kong); 5 Department of Brain Sciences, Imperial College London, London, United Kingdom; 6 School of Nursing, The Hong Kong Polytechnic University, Hong Kong, China (Hong Kong); 7 Institute of Active Ageing, The Hong Kong Polytechnic University, Hong Kong, China (Hong Kong); 8 School of Psychology and Clinical Language Sciences, University of Reading, Earley Gate, Whiteknights Campus, Reading, RG6 6ES, United Kingdom, 44 118 378 8523

**Keywords:** physical activity, physical exercise, exercising, gerontology, geriatric, older adult, older person, older people, active aging, postretirement work, cluster randomized controlled trial, RCT

## Abstract

**Background:**

Exercise interventions can reverse frailty. However, their scalability and sustainability are limited by manpower, which is reducing due to population aging. GrandMove is a program that combines healthy and productive aging strategies to (1) train and employ robust older adults as exercise coaches and (2) improve fitness and motivate the adoption of an exercise habit in older adults with frailty and prefrailty.

**Objective:**

The aim of this study is to examine the effectiveness of GrandMove in improving frailty, fitness, and quality of life in older adults with frailty and prefrailty.

**Methods:**

This cluster randomized controlled trial recruited older adults with frailty and prefrailty (N=390) living in the community. The 18-month exercise program consisted of three 6-month phases of lifestyle education (E), resistance exercise (R), and aerobic exercise (A). Each group of participants was randomized into 3 intervention sequence arms: the E-R-A group, the A-R-E group, and the R-A-E group.

**Results:**

At 6, 12, and 18 months, 346, 305, and 264 participants completed the frailty assessment, respectively. At 6 months, 100 of 346 participants (28.9%) were robust. A-R-E and R-A-E were no better than E-R-A as the active control in addressing frailty over the first 6 months (A-R-E: interaction coefficient 0.07, 95% CI −0.35 to 0.49, *P*=.68; R-A-E: interaction coefficient −0.02, 95% CI −0.42 to 0.38, *P*=.90). Compared to lifestyle education, resistance training and aerobic training over the first 6 months were associated with greater improvement in fitness measures of grip strength for the left hand (A-R-E: interaction coefficient 2.99, 95% CI 0.76 to 5.23, *P*=.009; R-A-E: interaction coefficient 2.21, 95% CI 0.63 to 4.36, *P*=.04) and right hand (A-R-E: interaction coefficient 3.75, 95% CI 1.54 to 5.97, *P*=.001; R-A-E: interaction coefficient 2.29, 95% CI 0.16 to 4.42, *P*=.04) and arm curl test (A-R-E: interaction coefficient 1.42, 95% CI 0.39 to 2.46, *P*=.007; R-A-E: interaction coefficient 1.11, 95% CI 0.12 to 2.11, *P*=.03). The sequence of exercise interventions (R-A-E vs A-R-E) did not make a difference in primary outcomes at 12 months, but the R-A-E group showed better quality of life (interaction coefficient 4.50, 95% CI 0.12 to 8.88, *P*=.008). Improved frailty outcomes were maintained by the end of the study, but the change in overall physical activity level was limited.

**Conclusions:**

Combining healthy and productive aging strategies is a scalable and sustainable way to improve frailty, fitness, and quality of life in older adults with frailty and prefrailty. Different combinations of lifestyle education and physical interventions improved frailty.

## Introduction

Frailty, a common condition that increases with age, is a dynamic clinical state that can improve or worsen over time [1,2]. About 1 in 10 older adults are frail and 46% are prefrail, a state with higher risk of progression to frailty [3]. Frailty is known to predict disability, mortality, risk of fractures, and many other adverse health outcomes [4]. To date, evidence for the management of frailty is the strongest with physical activity [5,6]. In older adults who are very frail, even small gains in strength can result in important functioning benefits and promote independence [5]. Evidence suggested superior outcomes with training carried out 3 or more times per week, for 30-45 minutes per session, and lasting at least 3-5 months [7]. Adherence to exercise training is good even in older adults with frailty, with few adverse events or safety concerns [5].

Practice guidelines widely recommend multicomponent exercise programs for the management of frailty [8]. Many programs concurrently prescribe multimodal exercise components, even though sequential prescription may promote adherence and minimize attrition [8,9]. The optimal sequence is yet to be investigated. Theoretically, strength and aerobic training contribute in different ways by slowing or compensating for muscle wasting and loss of endurance, as well as preventing other diseases and resisting the cascade of disability [10]. These exercise modalities impact on the older person’s quality of life by affecting their ability to lift load (eg, arising from a chair) and endurance in performing activities of daily living (eg, walking), allowing them to take control and lead a purposeful life. These may have implications for the design of exercise training protocols for frailty intervention.

Past development of exercise programs put a greater emphasis on maximizing physical benefits, while less focus was put on the scalability and sustainability of the program. It is foreseeable that the health care manpower gap as a result of population aging is increasing [11] and heavy reliance on professionally led formal health care services for addressing frailty is unviable and unsustainable. Our team developed a multicomponent exercise program, namely the GrandMove program, which has a group of trained coaches in their 50s to 60s deliver the interventions. Delivery of exercise interventions by peer coaches rather than health professionals is a key to the long-term scalability and sustainability of such programs in the context of population aging. Our program incorporated both healthy and productive aging strategies. Although the program had clear goals of improving frailty, fitness, and quality of life in older adults with frailty and prefrailty, our peer coaches took on their own path to successful aging by adopting an active lifestyle and engaging in postretirement paid work. A detailed description of the program design is provided in the Methods and Multimedia Appendix 1.

In this study, three intervention sequences were designed to (1) examine the effectiveness of a 6-month aerobic or resistance training program compared with lifestyle education as the active control intervention at 6 months, (2) investigate any order effect in resistance and aerobic training in older adults with frailty and prefrailty at 12 months, and (3) evaluate exercise habit formation over 18 months.

## Methods

### Study Design and Participants

This is an 18-month, multicenter, cluster randomized controlled trial with 3 intervention arms. A cluster design was used because of the logistical issues associated with the implementation of the interventions. The study sites included 14 community service centers for older adults and 15 public rental estates in Hong Kong. The staff from the participating sites referred potential participants based on their age and frailty status at screening.

### Ethical Considerations

Designated research assistants, who were not involved in the intervention, obtained written informed consent and conducted screening interviews with the participants. The study protocol was approved by the institutional ethics committee (reference number EA1511048). The study was registered with the HKU Clinical Trials Registry (reference number HKUCTR-1964). All data used in this study were deidentified before analysis. Participants did not receive any compensation for their involvement.

### Participants

Target study participants were older adults living in the community who did not have any contradictions to participation in a moderate level of physical activity. Between February 2016 and May 2017, older adults were invited to take part in this study if they were aged 65 years or older and were screened as either prefrail (scores of 1‐2 on the FRAIL [Fatigue, Resistance, Ambulation, Illness, and Loss of weight] scale) or frail (scores of 3‐5 on the FRAIL scale) [2]. To ensure the safety of the participants, older adults were excluded from participating if they reported having specific conditions. Specific exclusion criteria are described in Multimedia Appendix 2.

The sample size was calculated based on an estimated 0.5-point improvement in the FRAIL score (SD 1) between an exercise condition versus lifestyle education, using a 2-sided test at 1% significance level with 80% power. Assuming a 20% dropout rate, the minimum sample size is 120 per arm. It was estimated that about 1 in 10 older adults was frail. To allow subgroup analysis by frailty status, the ratio of participants with prefrailty to those with frailty was set at about 6:4 (Multimedia Appendix 2).

### Randomization and Masking

As shown in Multimedia Appendix 3, each group of participants was randomly assigned into one of three parallel arms with different intervention sequences: (1) lifestyle education – resistance training – aerobic training (E-R-A); (2) aerobic training – resistance training – lifestyle education (A-R-E); and (3) resistance training – aerobic training – lifestyle education (R-A-E). Each intervention component lasted for 6 months, totaling up to 18 months. The sequence of the intervention components was designed to address the three research aims: (1) to evaluate the effectiveness of aerobic and resistance training at 6 months compared to lifestyle education, (2) to investigate the order effect of aerobic and resistance training at 12 months, and (3) to evaluate exercise habit formation over 18 months.

Randomization (1:1:1) was done by a research assistant using computer-generated random numbers stratified by frailty status. The person who was responsible for generating the random allocation sequence was not involved in any other parts of the research. Each group of consecutive participants was assigned to one of the 3 arms. Group allocation could not be masked for persons delivering or receiving the interventions. Our program exercise physiologists, who were responsible for developing the exercise protocols, training exercise coaches, and conducting the fitness tests with participants, were blind to the group assignment. Research assistants designated to assess outcomes were blind to group assignment and had no involvement in the delivery of interventions.

### Intervention Program: GrandMove

The GrandMove program is a structured exercise training program that contains 2 protocols, one focusing on aerobic exercise and the other on resistance exercise. Two protocols for aerobic training and resistance training were designed by our program exercise physiologists with certified training in strength and conditioning and tailored for older adults with prefrailty and frailty in Hong Kong. Robust older people were trained as exercise coaches to deliver the exercise protocols. The design of the program was guided by social learning theory [12] and behavioral principles [13] (see Multimedia Appendix 1 for program characteristics). Both protocols were designed to be workable in a small home or group, using only small training implements such as rubber bands, towels, and water bottles for exercising. Each protocol had 5 levels, which indicate different levels of intensity. Based on the initial assessment, participants would start at a level that was realistic and attainable. A participant who reached a standard of fitness and strength in that level would continue with the next level.

The lifestyle education condition was comprised of 12 group sessions of health talks and 36 telehealth sessions. All health talks that covered different topics were delivered in a small group format by a retired nurse. Telehealth sessions involved a research assistant reviewing the health topics with participants and consolidating their understanding over the phone.

Each exercise intervention component lasted for 6 months (Multimedia Appendix 4). The 6-month schedule was designed to provide active coaching in the first 3 months, followed by monitoring and supervision (4th and 5th month), and self-sustained practice (6th month). Group sessions were provided in a small group format (8‐10 older adults per group led by 2 coaches). Lifestyle education was designed to match the frequency of contacts with the exercise interventions.

### Measures

The participants were assessed at baseline, 6 months, 12 months, and 18 months. Each assessment included a battery of self-report instruments administered through a structured interview and physical tests. Basic demographic characteristics were obtained, including age, gender, education level, marital status, and living arrangement.

### Primary Outcomes

There were 3 primary outcomes. First, the frailty score was calculated using the 5-item FRAIL scale [2]. The items cover areas of fatigue, resistance, aerobic fitness, illnesses, and loss of weight. FRAIL scores are classified into three categories: robust (score of 0), prefrail (score of 1-2), and frail (score of 3-5). Second, physical performance was measured using the Short Physical Performance Battery (SPPB) [14]. The SPPB score ranges from 0‐12, with a higher score indicating better performance. Third, quality of life was measured using the World Health Organization Quality of Life – Older adults module (WHOQoL-OLD) scale validated in a Chinese population [15]. The total score ranges from 0‐100, with a higher score indicating better quality of life.

### Secondary Outcomes

There were 3 fitness performance measures including isometric handgrip strength as measured by a digital hand dynamometer (Jamar Plus+), the 30-second arm curl test [16], and the 2-minute step test [17]. The hand grip strength of each hand was the average score of 3 trials. Higher fitness scores indicate better performance.

Other secondary outcomes included instrumental activities of daily living (IADL) as measured using the Lawton IADL scale (score range 0‐100, with higher scores indicating a lower level of disability) [18], level of physical activity as measured using the Physical Activity Scale for the Elderly (PASE; higher scores indicate a higher level of physical activity) [19], sleep quality as measured using the Pittsburgh Sleep Quality Index (PSQI; higher scores indicate worse overall sleep quality) [20], social functioning as measured using the Lubben Social Network Scale (LSNS; higher scores indicate a greater level of social support) [21], and depressive symptoms as measured using the Patient Health Questionnaire-9 (PHQ-9; score range 0‐27, with higher scores indicating greater symptom severity) [22].

### Statistical Analysis

We conducted all analyses on an intention-to-treat basis. We generated descriptive statistics for baseline characteristics. The proportions of participants who achieved a robust or an improved frailty status (from frail to prefrail/robust or from prefrail to robust) at each follow-up were described. Within-group changes in proportions of robust or improved frailty outcome were tested using mixed effect logistic regression models.

The difference in outcome measures between intervention groups was estimated at each follow-up time point using repeated measures mixed effect linear regression models, adjusting for the fixed effects of gender and age at baseline. The main analysis using mixed models in the whole sample will further adjust for other baseline covariates that were found to be significantly different between groups. To examine the effectiveness of 6-month aerobic or resistance training, outcomes in the A-R-E group and R-A-E group were compared to the E-R-A group. To investigate the order effect in aerobic and resistance training, we compared 12-month outcomes between the R-A-E group and the A-R-E group (reference group). To evaluate the maintenance of exercise effect and habit formation, we compared the outcomes including the level of physical activity between groups at 18 months. As intervention components were switched at the 6th and 12th month, time was treated as a categorical variable instead of continuous to account for the possible nonlinear relationship between time and outcome variables. Treatment effect referred to the coefficient of the intervention group × time interaction. Subgroup analyses by baseline frailty status and gender were individually made to delineate the differential treatment effects. Since 12 outcomes were examined in this study, we set the significance level at 1% and reported the 99% CIs for the primary outcomes to account for multiple comparison bias. The significance level for the secondary outcomes was set at 5%. All analyses were conducted using Stata/MP (version 17.0; StataCorp LLC).

## Results

### Sample Characteristics

**Figure 1.**
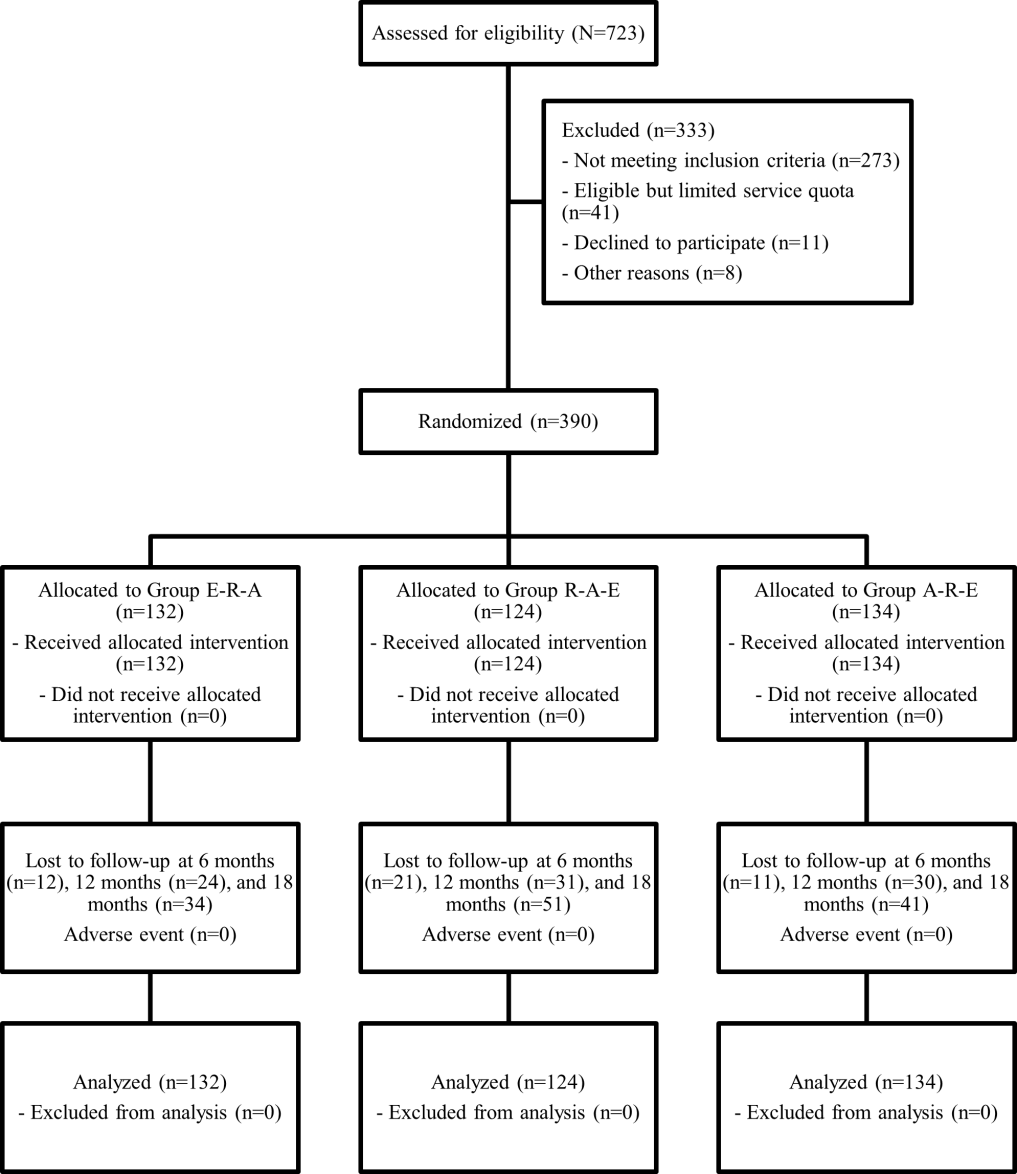
Study flow chart. All participants were analyzed in repeated measures mixed-effect regression models.

In total, 723 potential participants were assessed for eligibility, of whom 390 (53.9%) were randomized (Figure 1). Of the 390 participants included in the study, 132 (33.8%) were assigned to the E-R-A group, 124 (31.8%) to A-R-E, and 134 (34.4%) to R-A-E.

Baseline characteristics for participants by intervention group are shown in Table 1. The average FRAIL score, SPPB score, and overall quality of life at baseline were 2.1 (SD 0.9), 7.8 (SD 2.8), and 91.6 (SD 13.1), respectively. Characteristics of participants in the 3 groups were similar, except for the quality of life (*F*_2,385_=3.26, *P*=.04) and arm curl test scores (*F*_2,382_=5.47, *P*=.005). As suggested by the mean PASE scores, all 3 groups were considered as having low activity levels in general [23]. No reports of adverse events that led to death, hospitalization, or medical attendance were received by the end of the program.

**Table 1.** Baseline characteristics of the study population (n=390).

	E^a^-R^b^-A^c^ group (n=132)	A-R-E group (n=124)	R-A-E group (n=134)	*F*/χ^2^ (df)	*P* value
Age (years), mean (SD)	80.5 (7.5)	80.9 (7.1)	81.2 (7.4)	*F*=0.25 (2, 387)	.78
Gender, female, n (%)	106 (80.3)	97 (78.2)	105 (78.4)	χ^2^=0.21 (2)	.90
**Education, n (%)**				χ^2^=6.15 (6)	.41
No formal education	79 (59.9)	66 (53.2)	73 (54.5)		
Primary school	27 (20.5)	29 (23.4)	30 (22.4)		
Junior middle school	11 (8.3)	10 (8.1)	19 (14.2)		
High school or above	15 (11.4)	19 (15.3)	12 (9)		
**Marital status, n (%)**				χ^2^=2.04 (4)	.73
Married	47 (35.6)	49 (40.2)	45 (33.8)		
Widowed	75 (56.8)	62 (50.8)	79 (59.4)		
Others	10 (7.6)	11 (9)	9 (6.8)		
**Living alone**	38 (28.8)	46 (37.4)	47 (35.3)	χ^2^=2.34 (2)	.31
**Frailty status, n (%)**				χ^2^=0.70 (2)	.70
Prefrail	80 (60.6)	80 (64.5)	80 (59.7)		
Frail	52 (39.4)	44 (35.5)	54 (40.3)		
5-item FRAIL^d^ scale, mean (SD)	2.2 (0.9)	2.1 (0.9)	2.1 (1)	*F*=0.27 (2, 387)	.76
SPPB^e^, mean (SD)	8 (2.7)	7.5 (2.9)	7.8 (2.9)	*F*=1.14 (2, 387)	.32
WHOQoL-OLD^f^, mean (SD)	93.5 (12.3)	91.9 (12.4)	89.4 (14.1)	*F*=3.26 (2, 385)	.04
Grip strength (left hand), mean (SD)	34.5 (11.5)	33.6 (12.7)	33 (14.6)	*F*=0.46 (2, 381)	.63
Grip strength (right hand), mean (SD)	36.2 (12.6)	33.7 (13)	35.7 (14.9)	*F*=0.06 (2, 382)	.94
30-s arm curl test, mean (SD)	12.1 (4.3)	10.6 (4.3)	10.5 (4.1)	*F*=5.47 (2, 382)	.005
2-min step test, mean (SD)	69.2 (28.2)	65.3 (28.2)	66.6 (30.8)	*F*=0.60 (2, 382)	.55
IADL^g^, mean (SD)	14.8 (2.7)	15.2 (3.1)	15.2 (2.7)	*F*=0.99 (2, 385)	.37
PASE^h^, mean (SD)	70.6 (41.9)	73.1 (41.7)	72.3 (47.7)	*F*=0.11 (2, 382)	.90
LSNS^i^, mean (SD)	21.1 (9.6)	21.8 (9.5)	21.4 (9.7)	*F*=0.14 (2, 385)	.87
PSQI^j^, mean (SD)	8.3 (4.3)	8.1 (3.9)	8.3 (3.7)	*F*=0.10 (2, 356)	.91
PHQ-9^k^, mean (SD)	4 (4.9)	3.8 (4.7)	3.3 (4.5)	*F*=0.86 (2, 384)	.42

a E: lifestyle education.

b R: resistance training.

c A: aerobic training.

d FRAIL: Fatigue, Resistance, Ambulation, Illness, and Loss of weight.

e SPPB: Short Physical Performance Battery.

f WHOQoL-OLD: Cantonese version of the World Health Organization Quality of Life – Older adults module.

g IADL: Lawton’s Instrumental Activities of Daily Living scale.

h PASE: Physical Activity Scale for the Elderly.

i LSNS: Lubben Social Network Scale.

j PSQI: Pittsburgh Sleep Quality Index.

k PHQ-9: Patient Health Questionnaire-9.

### Change in Frailty Status

**Figure 2.**
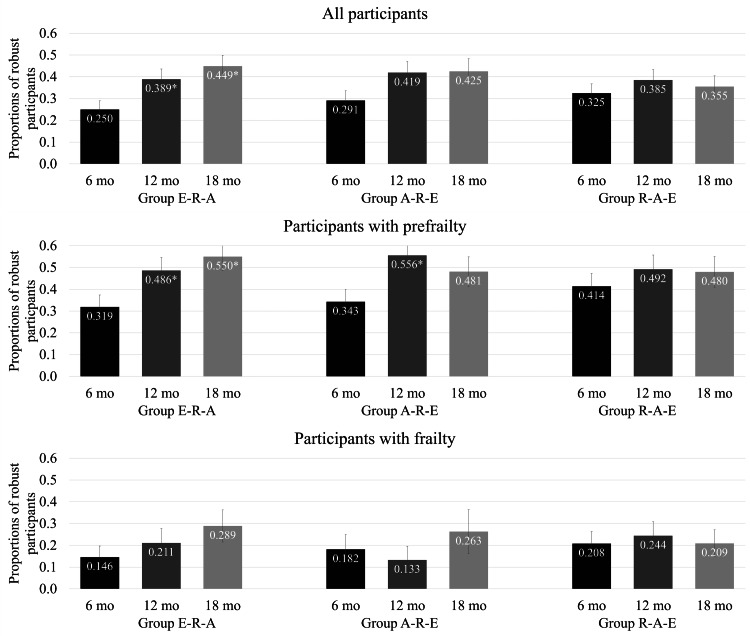
Percentage of older participants obtaining robust status at the follow-up assessment time points. Absolute numbers are reported in Multimedia Appendix 5. The intervention only targeted older adults with frailty and prefrailty, and thus no robust participants were included at baseline. Asterisks (*) indicate a further significant increase (*P*<.05) in the proportion of robust status from 6 months. A: aerobic training; E: lifestyle education; R: resistance training.

We found a substantial overall improvement in frailty status (Figure 2 and MultimediaAppendices 5  6), both in terms of achieving a robust status and an improved status (from frail to prefrail/robust or from prefrail to robust).

In the prefrail sample, 35.8% (76/212) of all participants became robust after 6 months. The proportion of robust participants increased to 51.0% (98/192) at 12 months and remained stable (83/164, 50.6%) at 18 months. In the frail sample, 17.9% (24/134) of participants became robust at 6 months. The proportion increased to 20.4% (23/113) at 12 months and continually increased to 25% (25/100) at 18 months. In terms of within-group changes, participants from the E-R-A group and A-R-E group (prefrail sample only) continued to have a significant improvement in frailty beyond 6 months (Figure 2 and MultimediaAppendices 5  6). Although a smaller proportion of participants with frailty obtained a robust status within the first 6 months, the majority of them progressed to prefrailty.

### Aerobic Versus Resistance Training: 6-Month Outcomes

As shown in Table 2, there was an overall improvement (having a significant time effect) in FRAIL score (coefficient −0.81, 95% CI −1.09 to –0.52; *P*<.001), IADL (coefficient 0.48, 95% CI 0.04 to 0.91; *P*=.03), PSQI (coefficient −0.74, 95% CI −1.34 to –0.14; *P*=.02), and PHQ-9 (coefficient −0.98, 95% CI −1.81 to −0.16; *P*=.02) in all groups over 6 months, suggesting that participants had improved frailty and functional and psychological outcomes.

**Table 2.** Summary of time and group × time interaction effects on primary and secondary outcomes (whole sample).

	Baseline to 6 months		Baseline to 12 months		Baseline to 18 months	
Coefficient (95% or 99% CI)	*P* value	Coefficient (95% or 99% CI)	*P* value	Coefficient (95% or 99% CI)	*P* value
**5-item FRAIL**^a^ **scale**						
Time effect	−0.81 (−1.09 to −0.52)	<.001	−1.10 (−1.40 to −0.80)	<.001	−1.06 (−1.36 to −0.75)	<.001
A-R-E^b^ group × time	0.07 (−0.35 to 0.49)	.68	0.16 (−0.27 to 0.60)	.33	0.16 (−0.29 to 0.62)	.35
R-A-E group × time	−0.02 (−0.42 to 0.38)	.90	0.25 (−0.17 to 0.67)	.13	0.20 (−0.23 to 0.64)	.23
**SPPB** ^c^						
Time effect	−0.22 (−0.66 to 0.31)	.20	−0.61 (−1.08 to −0.15)	.003	−0.64 (−1.11 to −0.16)	.002
A-R-E group × time	0.15 (−0.51 to 0.80)	.57	0.27 (−0.41 to 0.95)	.31	0.34 (−0.25 to 1.34)	.22
R-A-E group × time	0.03 (−0.60 to 0.65)	.91	0.04 (−0.62 to 0.70)	.88	−0.22 (−0.90 to 0.46)	.52
**WHOQoL-OLD** ^d^						
Time effect	0.22 (−2.62 to 3.07)	.84	1.66 (−1.31 to 4.63)	.15	0.52 (−2.53 to 3.57)	.66
A-R-E group × time	3.34 (−0.85 to 7.52)	.04	−0.73 (−5.10 to 3.63)	.66	1.85 (−2.75 to 6.45)	.30
R-A-E group × time	5.23 (1.21 to 9.24)	.001	3.77 (−0.47 to 8.01)	.02	4.13 (−0.23 to 8.49)	.02
**Grip strength (left)**						
Time effect	−2.81 (−4.31 to −1.30)	<.001	−0.22 (−1.81 to 1.36)	.78	Not reported
A-R-E group × time	2.99 (0.76 to 5.23)	.009	0.20 (−2.14 to 2.54)	.87		
R-A-E group × time	2.21 (0.63 to 4.36)	.04	−0.55 (−2.82 to 1.72)	.63		
**Grip strength (right)**						
Time effect	−3.12 (−4.62, to –1.63)	<.001	−1.12 (−1.12 to 0.45)	.16	Not reported	
A-R-E group × time	3.75 (1.54 to 5.97)	.001	1.06 (−1.25 to 3.38)	.37		
R-A-E group × time	2.29 (0.16 to 4.42)	.04	−0.50 (−2.75 to 1.75)	.66		
**30-s arm curl**						
Time effect	−0.77 (−0.78 to 0.63)	.83	0.12 (−0.62 to 0.85)	.76	0.12 (−0.63 to 0.88)	.75
A-R-E group × time	1.42 (0.39 to 2.46)	.007	1.46 (0.38 to 2.54)	.008	0.52 (−0.63 to 1.66)	.38
R-A-E group × time	1.11 (0.12 to 2.11)	.03	1.03 (−0.02 to 2.09)	.054	0.65 (−0.44 to 1.73)	.24
**2-min step test**						
Time effect	2.75 (−1.81 to 7.31)	.24	8.46 (3.69 to 13.24)	.001	7.99 (2.99 to 12.85)	.002
A-R-E group × time	5.51 (−1.23 to 12.25)	.11	2.49 (−4.57 to 9.54)	.49	1.10 (−6.32 to 8.52)	.77
R-A-E group × time	1.38 (−5.09 to 7.84)	.68	−3.17 (−9.99 to 3.66)	.36	−4.46 (−11.50 to 2.59)	.22
**IADL** ^e^						
Time effect	0.48 (0.04 to 0.91)	.03	0.64 (0.19 to 1.09)	.006	0.74 (0.27 to 1.20)	.002
A-R-E group × time	−0.32 (−0.95 to 0.32)	.33	-0.77 (−1.43 to −0.11)	.02	-0.88 (−1.59 to −0.18)	.01
R-A-E group × time	−0.38 (−0.99 to 0.24)	.23	−1.09 (−1.74 to 0.45)	.001	−1.22 (−1.89 to −0.56)	<.001
**PASE** ^h^						
Time effect	2.25 (−6.18 to 10.67)	.60	3.11 (−5.67 to 11.89)	.49	10.99 (2.06 to 19.90)	.02
A-R-E group × time	4.65 (−7.70 to 17.00)	.46	0.37 (−12.45 to 13.19)	.96	−3.29 (−16.72 to 10.14)	.63
R-A-E group × time	2.32 (−9.59 to 14.24)	.70	0.88 (−11.60 to 13.36)	.89	−3.00 (−15.76 to 9.77)	.64
**LSNS** ^f^						
Time effect	0.89 (−0.66 to 2.44)	.26	2.43 (0.81 to 4.06)	.003	1.39 (−0.27 to 3.06)	.10
A-R-E group × time	−1.73 (−4.02 to 055)	.14	−1.82 (−4.21 to 0.56)	.13	−0.68 (−3.20 to 1.83)	.60
R-A-E group × time	−0.48 (−2.67 to 1.72)	.67	−1.28 (−3.60 to 1.04)	.28	0.23 (−2.15 to 2.62)	.85
**PSQI** ^g^						
Time effect	−0.74 (−1.34 to −0.14)	.02	−0.96 (−1.59 to −0.33)	.003	0.17 (−0.48 to 0.81)	.61
A-R-E group × time	0.47 (−0.43 to 1.36)	.31	1.16 (0.23 to 2.09)	.02	−0.31 (−1.29 to 0.68)	.54
R-A-E group × time	0.58 (−0.28 to 1.44)	.19	0.94 (0.03 to 1.85)	.04	0.64 (−0.29 to 1.57)	.18
**PHQ-9** ^i^						
Time effect	−0.98 (−1.81 to −0.16)	.02	−1.14 (−2.00 to −0.27)	.01	−1.19 (−2.38 to −0.61)	.001
A-R-E group × time	0.43 (−0.79 to 1.65)	.49	0.49 (−0.78 to 1.76)	.45	0.69 (−0.65 to 2.03)	.31
R-A-E group × time	1.34 (0.17 to 2.52)	.02	1.51 (0.27 to 2.75)	.02	2.14 (0.87 to 3.41)	.001

a FRAIL: Fatigue, Resistance, Ambulation, Illness, and Loss of weight.

b A: aerobic training, R: resistance training, E: lifestyle education.

c SPPB: Short Physical Performance Battery.

d WHOQoL-OLD: Cantonese version of the World Health Organization Quality of Life - Older adults module.

e IADL: Lawton’s Instrumental Activities of Daily Living scale.

f LSNS: Lubben Social Network Scale.

g PASE: Physical Activity Scale for the Elderly.

h PSQI: Pittsburgh Sleep Quality Index.

i PHQ-9: Patient Health Questionnaire-9.

j Measurements reporting 99% CI: 5-item FRAIL scale, SPPB, and WHOQoL-OLD. All other scales used 95% CI.

At 6 months, participants with resistance training (R-A-E group) compared with those with lifestyle education (E-R-A group) had greater improvement in quality of life (interaction coefficient 5.23, 99% CI 1.21 to 9.24; *P*<.001) but not in FRAIL score and SPPB. Similar findings were observed in the prefrail subsample (Multimedia Appendix 7). Aerobic training (A-R-E group) was not different from lifestyle education in the primary outcomes.

For the secondary outcomes, both aerobic training (A-R-E group) and resistance training (R-A-E group) performed better in several fitness outcomes than the E-R-A group. The A-R-E group was associated with greater improvement in left hand grip strength (interaction coefficient 2.99, 95% CI 0.76 to 5.23; *P*=.009), right hand grip strength (interaction coefficient 3.75, 95% CI 1.54 to 5.97; *P*=.001), and the arm curl test (interaction coefficient 1.42, 95% CI 0.39 to 2.46; *P*=.007) than lifestyle education (E-R-A group). The R-A-E group was also associated with greater improvement in the 3 outcomes (Table 2). The A-R-E and R-A-E groups did not differ from the E-R-A group in all other nonfitness measures including the 2-minute step test, IADL, PASE, LSNS, and PSQI over 6 months. The R-A-E group had a significantly higher level of PHQ-9 scores (interaction coefficient 1.34, 95% CI 0.17 to 2.52; *P*=.02). Results of the subgroup analysis by frailty status were summarized in MultimediaAppendices 7  8, whereas results of the subgroup analysis by gender were summarized in MultimediaAppendices 9  10.

### Aerobic and Resistance Training: The Order Effect (12-Month Outcomes)

To compare the order effect, we also compared the R-A-E group with the A-R-E group (reference group) on 12-month outcomes (Multimedia Appendix 11). The 2 groups did not differ in all outcomes except that the R-A-E group was associated with greater improvement in WHOQoL-OLD (interaction coefficient 4.50, 95% CI 0.12 to 8.88; *P*=.008). The result was similar when the analysis was applied in the prefrail subsample. Participants with frailty in the R-A-E group achieved significantly fewer steps in the 2-minute step test compared to their counterparts in the A-R-E group at 12 months (interaction coefficient −12.48, 95% CI −24.78 to −0.18; *P*=.047).

In sum, the findings suggested that the order of aerobic and resistance training did not have an impact on fitness outcome at 12 months, although some preliminary data suggested that undergoing resistance training before aerobic training might improve quality of life.

### Maintenance of Exercise Effect and Habit Formation

The A-R-E and R-A-E groups did not receive a physical intervention between 12 and 18 months. If exercise effects were maintained, we expected to observe gains to be maintained by the end of the study. As shown in Table 2, the improvement in WHOQoL-OLD seemed to be maintained at 18 months in these 2 groups compared to the E-R-A group but the *P* value did not reach the .01 threshold (interaction coefficient 4.13, 95% CI −0.23 to 8.49; *P*=.02).

However, the gains in IADL appeared to reduce in the A-R-E (interaction coefficient −0.88, 95% CI −1.59 to −0.18; *P*=.013) and the R-A-E groups (interaction coefficient −1.22, 95% CI −1.89 to −0.56; *P*<.001) and also for PHQ-9 in the R-A-E group (interaction coefficient 2.14, 95% CI 0.87 to 3.41; *P*=.001).

Neither aerobic training nor resistance training improved PASE at 6 months compared to lifestyle education. We observed a significant time effect on PASE over 18 months in the whole sample (coefficient 10.99, 95% CI 2.06 to 19.90; *P*=.02) and in the frail subsample (coefficient 14.6, 95% CI 1.39 to 27.81; *P*=.03), indicating that combined exercise and lifestyle education, regardless of intervention sequence, might improve individuals’ level of physical activity.

## Discussion

### Principal Findings

This study examined the effectiveness of both aerobic training and resistance training using a sequential cluster randomized controlled trial design, which enabled us to examine how these physical activity interventions and their sequence might influence frailty outcomes. Although lifestyle education was initially added as a comparator intervention, our findings have demonstrated that the 18-month intervention combining lifestyle education and physical interventions is a viable strategy to address frailty and is health-promoting. The frailty score improved across groups, providing further evidence that physical frailty is reversible. Our study also produced a few other key findings. Aerobic training or resistance training tended to improve fitness performance but was not superior to an intensive lifestyle education program in addressing frailty. It is possible that 6 months of resistance training followed by another 6 months of aerobic training might be better at improving quality of life. Although the overall improvement in frailty outcome was maintained over 18 months, IADL and PHQ-9 appeared to worsen over the post–physical intervention period. We observed a small increase in the level of physical activity over 18 months, but its relation to the formation of exercise habits and an active lifestyle is yet to be determined.

Despite physical activity intervention being the most widely studied and recommended approach for the management of frailty [24], we found that a single-mode exercise program (aerobic or resistance) for 6 months was no better than lifestyle education in addressing frailty. The findings coincided with a recent systematic review suggesting that physical activity intervention, when compared with an active control intervention, was not associated with a significant reduction in frailty [25]. We found that the administration of 2 physical interventions in sequence did not further improve frailty scores but participants were able to maintain the gain accrued over the first 6 months. The 3 study arms approximated a multifaceted intervention that incorporated physical, psychosocial, and educational components. There were a few multifaceted studies [26-28] but cross-study comparison was difficult due to their different designs and the lack of frailty as an outcome. The Hatoyama Cohort Study [27] found that resistance exercise in combination with nutritional education and psychosocial programs for 3 months successfully reduced the prevalence of frailty by 24%. Our study further added that the sequence of intervention components, if not delivered concurrently, might not make a significant difference. Nonetheless, older adults with frailty may find the program more acceptable if they have greater control over the sequence of interventions.

We added to the literature that psychosocial intervention might be complementary to physical activity intervention for the management of frailty. Following the lifestyle education program, the subsequent addition of physical intervention appeared to generate further gain. Participants from the E-R-A group were observed to have continued improvement in frailty outcomes after switching from lifestyle education to resistance training. There were only a few known studies to date exploring the effectiveness of psychosocial interventions on frailty. A Swedish study evaluated a 4-week senior group program similar to our lifestyle education program, which introduced and discussed various healthy lifestyle topics [29]. The senior group program had no impact on frailty outcomes, but it was reported to delay activities of daily living deterioration. Our lifestyle education program was more intensive in terms of frequency and duration than the senior group program. Further studies may explore whether shorter or longer combined interventions may make a difference in frailty outcomes.

Since frailty is suggested to result from cumulative declines in multiple physiologic systems [1], it is not surprising that interventions targeting different systems may yield similar positive results (ie, the equifinality principle). There might be common factors across the intervention approaches that mitigate frailty, such as social support from peers (exercise coaches and retired nurses). In our study, all 3 intervention components encouraged social engagement through the group sessions and peer coaching. Previous studies have suggested that social support might directly or indirectly contribute to higher levels of physical activity in older adults [30-32] and could therefore be health-promoting and lead to a healthier lifestyle. Further studies should include a comparison group without social influence and a measure of perceived social support to allow for an estimation of its impact on frailty and quality of life. Future studies may also include measures of other psychological mediators such as self-efficacy, enjoyment, and the use of behavioral and cognitive processes [33] for mediation analyses.

The subgroup analysis by frailty status showed that participants with frailty appeared to have a greater magnitude of reduction in FRAIL score, further demonstrating that frailty is a modifiable condition. The frail subgroup also had consistently reported increased social engagement and some reduction in depressive symptoms over 18 months. Some improvements in physical fitness were observed but may not have been sustained beyond the active intervention period. The results echoed our hypothesis that active physical or psychosocial interventions might enhance social support for older adults at high risk of vulnerability and social isolation [34]. Physical activity guidelines should recommend older adults with frailty to engage in moderate-intensity exercise regularly [35]. The design of physical activity interventions should incorporate social or group elements that enhance social learning and reinforcement. With a sample predominantly comprised of female older adults (~80%), the results for the female subsample were largely consistent with those in the full sample. There may be a lack of power to detect significant intervention effects in the male subsample. These results should be interpreted with caution.

We observed a significant increase in the level of physical activity over 18 months, probably due in part to the significant change within the frail subsample. No evidence showed that participants with prefrailty increased their level of physical activity after interventions. It might take as long as 18 months to observe an overall increase in the level of physical activity. The potential of exercise and lifestyle interventions to modify long-term exercise habits remains unclear. It is common to observe a relapse pattern in health behavior once an intervention has ended [36]. Program effectiveness and acceptability are equally important as pain and discomfort associated with physical activity could be an obstacle to engaging in further physical activity [37]. In addition, a physical intervention combined with smartphone-assisted e-reminders, an activity tracker, and e-coaching may help habit formation [38]. It is equally important to implement strategies to discourage unhealthy habits simultaneously. It is possible that our lifestyle education program helped increase participants’ awareness about unhealthy habits and alternative healthy options in the environment. Further investigation to determine the optimal form of intervention(s) that best maintains exercise habit formation is warranted.

We consider the delivery of exercise interventions by peer coaches to be the key to long-term scalability and sustainability in the context of population aging. More research is needed to formally evaluate the sustainability and cost-effectiveness of the intervention model. Future research should consider the productive aging component for engaging retired older adults in productive activities, task shifting from health professionals to more available human resources (ie, peer coaches), and the implications of an increased number of robust older people in the community. Health care utilization associated with a positive change in frailty outcomes has not been explored, but it is worth further investigation.

### Limitations

Due to the complexity of the study design, we lacked a care-as-usual comparator group. The active control group only abstained from physical intervention in the first 6 months. Therefore, we were unable to evaluate the effectiveness of physical activity interventions for 12 months compared with an active control intervention. Further multifaceted intervention studies may include both psychoeducational components in combination with physical interventions. An accelerometer-based measure of physical activity level may be better than a self-reported one in estimating the effect of a physical intervention on habit formation [39]. Similar to other physical intervention programs, the generalization of results might be limited by attrition bias and a predominantly female sample. This program was tested in a densely populated community. This setting may enhance the viability and cost-effectiveness of delivering both group and home sessions via peer coaches. Adaptations may be needed if the model is applied in other contexts, for example, in rural areas, which will require a separate evaluation of feasibility and effectiveness.

### Conclusions

In contrast to most previous trials, we attempted to address frailty by examining physical interventions in comparison with a novel comparator, that is, lifestyle education. Both tested physical interventions and lifestyle education were effective in improving frailty status. A simultaneous improvement in quality of life was observed. Participants with frailty appeared to benefit beyond a frailty outcome. Despite the positive findings, the impact of the interventions on exercise habit formation is yet to be investigated.

## Supplementary material

10.2196/65636 Multimedia Appendix 1 Program design.

10.2196/65636 Multimedia Appendix 2 Additional information about study methods and attendance.

10.2196/65636 Multimedia Appendix 3 Components and sequence of the 3-arm trial.

10.2196/65636 Multimedia Appendix 4 Schedule for each intervention component.

10.2196/65636 Multimedia Appendix 5 Percentage of participants achieving robust status (from prefrail or frail to robust).

10.2196/65636 Multimedia Appendix 6 Percentage of older participants obtaining an improved frailty status at the follow-up assessment time points. The intervention only targeted older adults with prefrailty and frailty, and thus no robust participants were included at baseline. Asterisks (*) indicate a further significant increase (*P*<.05) in the proportion of improved frailty status from 6 months.

10.2196/65636 Multimedia Appendix 7 Summary of time and group × time interaction effects on primary and secondary outcomes (participants with prefrailty only).

10.2196/65636 Multimedia Appendix 8 Summary of time and group × time interaction effects on primary and secondary outcomes (participants with frailty only).

10.2196/65636 Multimedia Appendix 9 Summary of time and group × time interaction effects on primary and secondary outcomes (female participants only).

10.2196/65636 Multimedia Appendix 10 Summary of time and group × time interaction effects on primary and secondary outcomes (male participants only).

10.2196/65636 Multimedia Appendix 11 Comparison of outcomes between the R-A-E group and A-R-E group (reference) at the 12-month follow-up.

10.2196/65636 Checklist 1 CONSORT checklist. CONSORT: Consolidated Standards of Reporting Trials.

## Abbreviations

A-R-E: aerobic training - resistance training - lifestyle education
E-R-A: lifestyle education - resistance training - aerobic training
FRAIL: Fatigue, Resistance, Ambulation, Illnesses, and Loss of weight
IADL: Instrumental Activities of Daily Living
LSNS: Lubben Social Network Scale
PASE: Physical Activity Scale for the Elderly
PHQ-9: Patient Health Questionnaire-9
PSQI: Pittsburgh Sleep Quality Index
R-A-E: resistance training - aerobic training - lifestyle education
SPPB: Short Physical Performance Battery
WHOQoL-OLD: World Health Organization Quality of Life – Older adults module
