# Supplementary material for: Frailty, Fitness, and Quality of Life Outcomes of a Healthy and Productive Aging Program (GrandMove) for Older Adults With Frailty or Prefrailty: Cluster Randomized Controlled Trial
Source: JMIR Aging. 2025 May 14;8:e65636. doi: 10.2196/65636 (PMC12094531; doi:10.2196/65636)
Supplement: Multimedia Appendix 1 [file aging-v8-e65636-s001.docx]

**Multimedia Appendix 1. Program design**

| **Character-istics** | **Resistance Exercise** | **Aerobic Exercise** | **Lifestyle education** |
| --- | --- | --- | --- |
| Targets | Improve musculoskeletal system | Improve cardiorespiratory system | Enhance health knowledge and chronic illness management |
| Who delivered | 79 exercise coaches were trained to deliver the exercise interventions. To ensure their competence, all of them underwent 100-hour training (at least 80% attendance) and passed the test for administering the exercise protocols. The training covered basic exercise physiology, first aid (including cardiopulmonary resuscitation and bandaging), physical activity guidelines for older adults, protocol for progressive resistance exercise training, protocol for aerobic exercise training, motivation and coaching techniques, and principles for behavioral and lifestyle modification. They received regular supervision by exercise physiologists on the team. | | Retired nurses with years of working experience in chronic disease management. |
| Applied theories | - Social learning theory: Learn through direct observation of exercise coaches who are of similar age with a non-professional background. - Behavioral principles that target health behavior change and habit formation: Initiation of exercise behavior was supported by our well-defined exercise protocols for self-practice, event-based cues (i.e., joining the exercise training when they visit their center), minimal precondition concerning place and implements, and provision of reminders from the coach. Multiple reinforcing effects are expected to be generated from positive interaction with peers and coaches, increased sense of competency and accomplishments, positive verbal feedback from coach, and positive reinforcement as a result of improved health status. | | Health education |
| Format and interval (eTable 3) | Combined group and home practice, with phone follow-up. Active coaching (3 times/week) in the first three months, followed by monitoring and supervision (4^th^ and 5^th^ months) and self-sustained practice (6^th^ month) | | Group health talks or individual telehealth sessions for 3 times/week in the first 3 months, followed by two weekly telehealth sessions in the 4^th^ and 5^th^ months, and self-management of health and chronic conditions without active contact (6^th^ month) |
| Levels of difficulty | Five levels: Basic, Bronze, Silver, Gold, and Platinum elite. Participants started at a level that is realistic and provides enough challenge. Participants who reached a standard of fitness and strength in that level would continue with the next level. Although there will be individual difference in the pace of progression, the levels were designed to take on average 1 to 2 months to progress one level. | | Not applicable. |
| Protocol content and features | - Progression from stable support of bilateral to unilateral strength and balancing. - Each practice consisted of 11 exercise drills grouped into 3 cycles, with each drill to be performed 1 – 3 sets of 10 – 15 repetitions. - These cycles start with isolation exercise to compound exercise that uses several muscle groups collaboratively to perform a movement. - Can be carried out at home any time. | - Targeted to improve aerobic endurance up to 150 minutes of moderate level of aerobic training a week - Progression from sitting to standing exercises. - Each level consisted of 5 exercise drills paired with music. - Continuous exercise that allow elevation of heart rate to a level determined by perceived exertion.^a^ - Can be carried out at home any time. | Themes of 12 health talks, including self-management of chronic conditions; exercise; understanding and managing blood pressure; Understanding and managing diabetes; How to read food labels; Diet and nutrition (2 sessions); digestive health and constipation; sleep; pain; living with diseases; brain health |

^a^ Perceived exertion is measured using the Borg scale of self-perceived exertion (possible range of score, 6-20), with a level of “somewhat hard” indicating a level of moderate intensity training.
