# Supplementary material for: Frailty, Fitness, and Quality of Life Outcomes of a Healthy and Productive Aging Program (GrandMove) for Older Adults With Frailty or Prefrailty: Cluster Randomized Controlled Trial
Source: JMIR Aging. 2025 May 14;8:e65636. doi: 10.2196/65636 (PMC12094531; doi:10.2196/65636)
Supplement: Multimedia Appendix 2 [file aging-v8-e65636-s002.docx]

**Multimedia Appendix 2.** Additional information about study methods and attendance

| **Study methods and attendance** | **Description** |
| --- | --- |
| **Exclusion criteria** | To ensure the safety of the participants to participate in the exercise-based intervention according to original trial protocol, older adults were excluded if they had severe heart failure, uncontrolled angina, severe pulmonary disease, significant cognitive impairment, or end-stage disease with a life expectancy of less than 12 months. Older adults who had a history of stroke, hip fracture and myocardial infarction, or had undergone hip- or knee replacement surgery within the previous 6 months were also excluded. Significant cognitive impairment was defined as a clinical diagnosis of dementia or obtaining a score falling below the suggested dementia screening cut-off using the clock drawing test. |
| **Sample size calculation** | The sample size was calculated based on an estimated 0.5-point of improvement in the FRAIL score (SD = 1) between an exercise condition versus lifestyle education, using a two-sided test at 1% significance level with 80% power. It was estimated that the minimum sample size was 96, or 120 assuming 20% drop-out. Since there were more prefrail than frail older adults in the community, we purposively stratified the study sample by the frailty status so that the number of prefrail to frail participants in each intervention group was set at 6:4 (i.e., approximately 75 prefrail and 45 frail older adults in each group). |
| **Attendance** | For the E-R-A group, 61%, 53%, and 45% participants obtained at least 80% attendance in group sessions in the three intervention periods (i.e., first 6 months, 6-12 months, and 12-18 months), respectively. Respective statistics were 56%, 48%, and 26% for the A-R-E group; 64%, 48%, and 25% for the R-A-E group. Reasons of non-attendance included lack of motivation, time conflict, health problems, hospitalization, moving into residential care homes, and death. Overall attendance of individual home sessions was satisfactory (over 50% participants obtained at least 80% attendance). |
