# Supplementary figures and images for: Frailty, Fitness, and Quality of Life Outcomes of a Healthy and Productive Aging Program (GrandMove) for Older Adults With Frailty or Prefrailty: Cluster Randomized Controlled Trial
Source: JMIR Aging. 2025 May 14;8:e65636. doi: 10.2196/65636 (PMC12094531; doi:10.2196/65636)

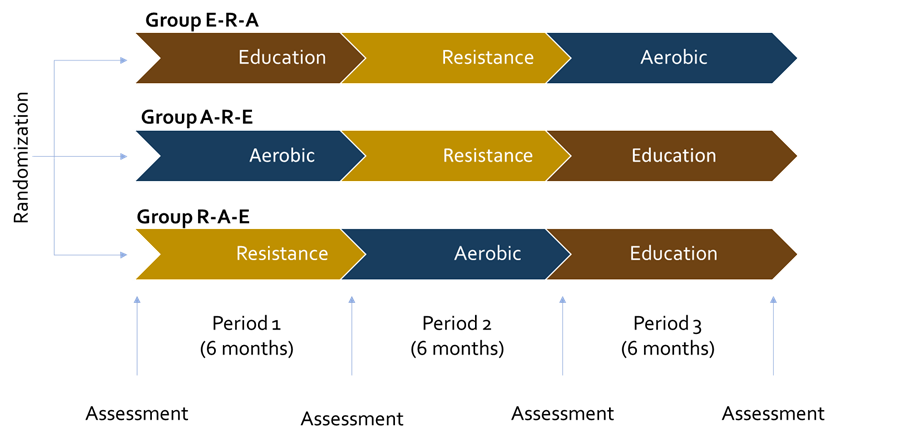

Supplement: Multimedia Appendix 3 [file aging-v8-e65636-s003.png]

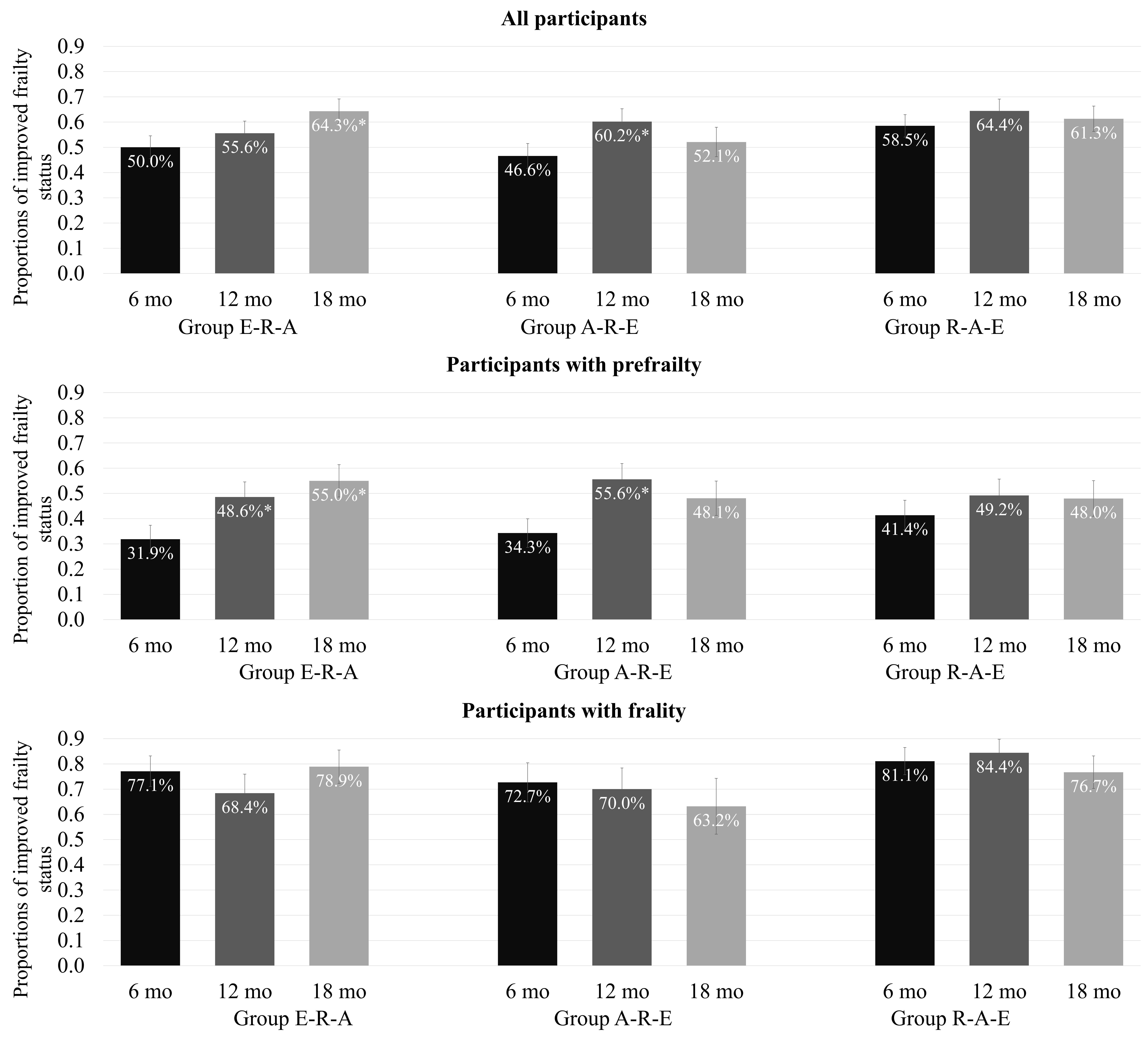

Supplement: Multimedia Appendix 6 [file aging-v8-e65636-s006.png]
