# Supplementary material for: Frailty, Fitness, and Quality of Life Outcomes of a Healthy and Productive Aging Program (GrandMove) for Older Adults With Frailty or Prefrailty: Cluster Randomized Controlled Trial
Source: JMIR Aging. 2025 May 14;8:e65636. doi: 10.2196/65636 (PMC12094531; doi:10.2196/65636)
Supplement: Multimedia Appendix 4 [file aging-v8-e65636-s004.docx]

**Multimedia Appendix 4.** Schedule for each intervention component

| **Stages** | **Aerobic / Resistance Exercise** | **Lifestyle education** |
| --- | --- | --- |
| 1^st^ month | Center-based group practice (1/week) and home practice with coach (2/week)^a^ | Center-based health talks (1/week) and telehealth sessions (2/week) |
| 2^nd^ month | Center-based group practice (1/week) and home practice with coach (1/week), phone call (1/week) | Center-based health talks (1/week) and telehealth sessions (2/week) |
| 3^rd^ month | Center-based group practice (1/week) and phone calls (2/week) | Center-based health talks (1/week) and telehealth sessions (2/week) |
| 4^th^ month | Phone calls (2/week) | Telehealth sessions (2/week) |
| 5^th^ month | Phone calls (1/week) | Telehealth sessions (1/week) |
| 6^th^ month | Weaning period | Weaning period |

^a^ Each group or home session will last for 1 hour.
