# Supplementary material for: Frailty, Fitness, and Quality of Life Outcomes of a Healthy and Productive Aging Program (GrandMove) for Older Adults With Frailty or Prefrailty: Cluster Randomized Controlled Trial
Source: JMIR Aging. 2025 May 14;8:e65636. doi: 10.2196/65636 (PMC12094531; doi:10.2196/65636)
Supplement: Multimedia Appendix 5 [file aging-v8-e65636-s005.docx]

**Multimedia Appendix 5.** Percentage of participants achieving robust status (from prefrail or frail to robust)

|  | Total sample | | Group  E-R-A | | Group  A-R-E | | Group  R-A-E | | Group difference | |
| --- | --- | --- | --- | --- | --- | --- | --- | --- | --- | --- |
|  | n/N | % | n/N | % | n/N | % | n/N | % | χ^2^ | P-  value |
| **Total sample** |  |  |  |  |  |  |  |  |  |  |
| **6 months** | 100/346 | 28.9 | 30/120 | 25.0^a^ | 30/103 | 29.1^a^ | 40/123 | 32.5^a^ | 1.69 | .43 |
| **12 months** | 121/305 | 39.7 | 42/108 | 38.9^a^ | 39/93 | 41.9 | 40/104 | 38.5 | 0.29 | .87 |
| **18 months** | 108/264 | 40.9 | 44/98 | 44.9 | 31/73 | 42.5 | 33/93 | 35.5 | 1.85 | .40 |
| **Prefrail sample** |  |  |  |  |  |  |  |  |  |  |
| **6 months** | 76/212 | 35.9 | 23/72 | 31.9 | 24/70 | 34.3 | 29/70 | 41.4 | 1.50 | .47 |
| **12 months** | 98/192 | 51.0 | 34/70 | 48.6 | 35/63 | 55.6 | 29/59 | 49.2 | 0.77 | .68 |
| **18 months** | 83/164 | 50.6 | 33/60 | 55.0 | 26/54 | 48.2 | 24/50 | 48.0 | 0.73 | .69 |
| **Frail sample** |  |  |  |  |  |  |  |  |  |  |
| **6 months** | 24/134 | 17.9 | 7/48 | 14.6 | 6/33 | 18.2 | 11/53 | 20.8 | 0.66 | .72 |
| **12 months** | 23/113 | 20.4 | 8/38 | 21.1 | 4/30 | 13.3 | 11/45 | 24.4 | 1.39 | .50 |
| **18 months** | 25/100 | 25.0 | 11/38 | 29.0 | 5/19 | 26.3 | 9/43 | 20.9 | 0.71 | .70 |

*Note.* A = Aerobic training. R = Resistance training. E = Lifestyle education.

^a^Significant improvement from the preceding measurement timepoint, i.e., from baseline to 6m, 6m to 12m, 12m to 18m (*P* < .05).
