# Supplementary material for: Frailty, Fitness, and Quality of Life Outcomes of a Healthy and Productive Aging Program (GrandMove) for Older Adults With Frailty or Prefrailty: Cluster Randomized Controlled Trial
Source: JMIR Aging. 2025 May 14;8:e65636. doi: 10.2196/65636 (PMC12094531; doi:10.2196/65636)
Supplement: Multimedia Appendix 7 [file aging-v8-e65636-s007.docx]

**Multimedia Appendix 7.** Summary of time and group × time interaction effects on primary and secondary outcomes (prefrail participants only)

|  | **Baseline to 6 months** | | **Baseline to 12 months** | | **Baseline to 18 months** | |
| --- | --- | --- | --- | --- | --- | --- |
|  | **Coefficient (95% or 9(% CI)** | **P-value** | **Coefficient (95% or 9(% CI)** | **P-value** | **Coefficient (95% or 9(% CI)** | **P-value** |
| **5-item FRAIL scale** |  |  |  |  |  |  |
| Time effect | -0.28 (-0.61, 0.05) | .031 | -0.77 (-1.11, -0.44) | <.001 | -0.67 (-1.02, -0.32) | <.001 |
| Group A-R-E * Time | -0.14 (-0.61, 0.34) | .452 | -0.03 (-0.51, 0.46) | .885 | 0.05 (-0.46, 0.56) | .802 |
| Group R-A-E * Time | -0.18 (-0.65, 0.30) | .335 | 0.34 (-0.15, 0.83) | .075 | 0.14 (-0.38, 0.65) | .490 |
| **SPPB** |  |  |  |  |  |  |
| Time effect | -0.13 (-0.75, 0.48) | .580 | -0.67 (-1.29, -0.04) | .006 | -0.5 (-1.15, 0.16) | .051 |
| Group A-R-E * Time | 0.5 (-0.38, 1.38) | .141 | 0.71 (-0.20, 1.62) | .045 | 0.26 (-0.68, 1.21) | .472 |
| Group R-A-E * Time | 0.21 (-0.67, 1.08) | .542 | 0.37 (-0.54, 1.28) | .293 | 0.14 (-0.82, 1.10) | .716 |
| **WHOQOL-OLD** |  |  |  |  |  |  |
| Time effect | 0.15 (-3.24, 3.55) | .907 | 1.65 (-1.78, 5.08) | .216 | 1.68 (-1.93, 5.28) | .231 |
| Group A-R-E * Time | 1.59 (-3.23, 6.42) | .395 | -0.76 (-5.70, 4.18) | .691 | -0.94 (-6.13, 4.26) | .643 |
| Group R-A-E * Time | 7.67 (2.83, 12.51) | <.001 | 6.35 (1.34, 11.35) | .001 | 5.37 (0.08, 10.68) | .009 |
| **Grip strength (left hand)** |  |  |  |  |  |  |
| Time effect | -2.2 (-3.92, -0.47) | .012 | -1.78 (-3.55, -0.01) | .049 | Not reported |  |
| Group A-R-E * Time | 1.98 (-0.49,4.46) | .116 | 1.12 (-1.47,3.70) | .398 |  |  |
| Group R-A-E * Time | 2.08 (-0.39,4.56) | .099 | 1.43 (-1.15,4.00) | .277 |  |  |
| **Grip strength (right hand)** |  |  |  |  |  |  |
| Time effect | -1.75 (-3.51,0.00) | .050 | -1.68 (-3.47,0.11) | .067 | Not reported |  |
| Group A-R-E * Time | 2.17 (-0.35,4.68) | .092 | 1.32 (-1.3,3.95) | .323 |  |  |
| Group R-A-E * Time | 0.97 (-1.56,3.49) | .453 | 0.23 (-2.39,2.85) | .863 |  |  |
| **30-sec arm curl** |  |  |  |  |  |  |
| Time effect | 0.01 (-0.90, 0.91) | .991 | 0.41 (-0.52, 1.34) | .390 | -0.02 (-0.99, 0.94) | .960 |
| Group A-R-E * Time | 1.92 (0.63, 3.22) | .004 | 1.09 (-0.25, 2.44) | .112 | -0.08 (-1.48, 1.32) | .911 |
| Group R-A-E * Time | 1.57 (0.28, 2.86) | .017 | 0.69 (-0.65, 2.04) | .313 | 0.87 (-0.55, 2.28) | .231 |
| **2-minute step test** |  |  |  |  |  |  |
| Time effect | 3.81 (-1.96, 9.58) | .196 | 5.01 (-0.88, 10.90) | .095 | 8.66 (2.47, 14.84) | .006 |
| Group A-R-E * Time | 9.09 (0.81, 17.36) | .031 | 7.65 (-1.00, 16.30) | .083 | 5.03 (-3.92, 13.97) | .270 |
| Group R-A-E * Time | 6.36 (-1.87, 14.59) | .130 | 4.71 (-3.85, 13.26) | .281 | 1.91 (-7.14, 10.96) | 0.679 |
| **IADL** |  |  |  |  |  |  |
| Time effect | 0.58 (0.04, 1.12) | .035 | 1.19 (0.65, 1.74) | <.001 | 1.07 (0.50, 1.65) | <.001 |
| Group A-R-E * Time | -0.31 (-1.08, 0.46) | .428 | -1.18 (-1.97, -0.39) | .003 | -0.84 (-1.67, -0.02) | .046 |
| Group R-A-E * Time | 0.21 (-0.56, 0.98) | .597 | -0.8 (-1.59, -0.00) | .050 | -0.83 (-1.68, 0.01) | .052 |
| **PASE** |  |  |  |  |  |  |
| Time effect | -4.25 (-15.39, 6.90) | .455 | 2.41 (-8.74, 13.57) | .672 | 10.87 (-0.79, 22.52) | .068 |
| Group A-R-E * Time | 16.23 (0.46, 32.01) | .044 | 3.95 (-12.11, 20.01) | .630 | -2.28 (-19.08, 14.52) | .790 |
| Group R-A-E * Time | 10.67 (-5.15, 26.48) | .186 | 3.32 (-12.94, 19.58) | .689 | 0.21 (-16.84, 17.26) | .981 |
| **LSNS** |  |  |  |  |  |  |
| Time effect | -1.35 (-3.33,0.62) | .180 | 1.39 (-0.60,3.39) | .171 | 0.41 (-1.69,2.51) | .701 |
| Group A-R-E * Time | 1 (-1.81,3.81) | .485 | -0.85 (-3.73,2.03) | .563 | 0.56 (-2.47,3.59) | .717 |
| Group R-A-E * Time | 1.99 (-0.83,4.80) | .167 | 0.64 (-2.28,3.56) | .668 | 2.99 (-0.10,6.08) | .058 |
| **PSQI** |  |  |  |  |  |  |
| Time effect | -0.73 (-1.49,0.03) | .058 | -0.73 (-1.50,0.03) | .060 | 0.42 (-0.38,1.22) | .306 |
| Group A-R-E * Time | -0.08 (-1.17,1.01) | .885 | 0.26 (-0.85,1.38) | .643 | -0.81 (-1.99,0.36) | .174 |
| Group R-A-E * Time | -0.3 (-1.40,0.80) | .595 | 0.42 (-0.72,1.57) | .466 | -0.56 (-1.76,0.65) | .364 |
| **PHQ-9** |  |  |  |  |  |  |
| Time effect | -0.76 (-1.80,0.28) | .152 | -1.28 (-2.33, -0.23) | .017 | -1.36 (-2.46, -0.26) | .016 |
| Group A-R-E * Time | -0.1 (-1.58,1.37) | .891 | -0.25 (-1.76,1.26) | .743 | 0.16 (-1.43,1.75) | .842 |
| Group R-A-E * Time | 0.21 (-1.28,1.69) | .785 | 0.45 (-1.09,1.99) | .567 | 0.58 (-1.04,2.21) | .481 |

*Note.* A = Aerobic training. R = Resistance training. E = Lifestyle education.

IADL = Lawton’s Instrumental Activities of Daily Living Scale; LSNS = Lubben Social Network Scale; PASE = Physical Activity Scale for the Elderly; PHQ-9 = Patient Health Questionnaire; PSQI = Pittsburgh Sleep Quality Index; SPPB = Short Physical Performance Battery; WHOQoL-OLD = Cantonese version of the World Health Organization Quality of Life - Older Adults Module.
