# Supplementary material for: Frailty, Fitness, and Quality of Life Outcomes of a Healthy and Productive Aging Program (GrandMove) for Older Adults With Frailty or Prefrailty: Cluster Randomized Controlled Trial
Source: JMIR Aging. 2025 May 14;8:e65636. doi: 10.2196/65636 (PMC12094531; doi:10.2196/65636)
Supplement: Multimedia Appendix 8 [file aging-v8-e65636-s008.docx]

**Multimedia Appendix 8.** Summary of time and group × time interaction effects on primary and secondary outcomes (frail participants only)

|  | **Baseline to 6 months** | | **Baseline to 12 months** | | **Baseline to 18 months** | |
| --- | --- | --- | --- | --- | --- | --- |
|  | **Coefficient (95% or 9(% CI)** | **P-value** | **Coefficient (95% or 9(% CI)** | **P-value** | **Coefficient (95% or 9(% CI)** | **P-value** |
| **5-item FRAIL scale** |  |  |  |  |  |  |
| Time effect | -1.53 (-1.97, -1.09) | **<.001** | -1.47 (-1.94, -0.99) | **<.001** | -1.67 (-2.15, -1.20) | **<.001** |
| Group A-R-E * Time | 0 (-0.68, 0.68) | .995 | 0.14 (-0.57, 0.85) | .609 | 0.23 (-0.55, 1.01) | .451 |
| Group R-A-E * Time | -0.04 (-0.65, 0.57) | .872 | -0.21 (-0.86, 0.44) | .411 | 0.17 (-0.49, 0.82) | .516 |
| **SPPB** |  |  |  |  |  |  |
| Time effect | -0.26 (-1.10, 0.57) | .412 | -0.45 (-1.36, 0.45) | .195 | -0.84 (-1.73, -0.05) | .015 |
| Group A-R-E * Time | 0.53 (-0.75, 1.82) | .286 | 0.7 (-0.65, 2.05) | .184 | 1.1 (-0.39, 2.59) | .056 |
| Group R-A-E * Time | 0.62 (-0.53, 1.76) | .167 | 0.34 (-0.89, 1.58) | .474 | 0.05 (-1.19, 1.28) | .923 |
| **WHOQoL-OLD** |  |  |  |  |  |  |
| Time effect | 0.02 (-4.85, 4.89) | .991 | 0.38 (-4.89, 5.65) | .852 | -1.98 (-7.25, 3.29) | .334 |
| Group A-R-E * Time | 8.45 (0.92, 15.98) | **.004** | 2.24 (-5.71, 10.18) | .468 | 7.87 (-0.91, 16.65) | .021 |
| Group R-A-E * Time | 2.54 (-4.20, 9.29) | .332 | 2.69 (-4.53, 9.91) | .337 | 3.55 (-3.72, 10.82) | .208 |
| **Grip strength (left hand)** |  |  |  |  |  |  |
| Time effect | -3.95 (-6.69, 1.22) | **.005** | 2.7 (-0.29, 5.68) | **.000** | Not reported |  |
| Group A-R-E * Time | 7.42 (3.19, 11.66) | **.001** | 0.91 (-3.55, 5.37) | .688 |  |  |
| Group R-A-E * Time | 4.22 (0.46, 7.98) | **.028** | -2.63 (-6.61, 1.54) | .223 |  |  |
| **Grip strength (right hand)** |  |  |  |  |  |  |
| Time effect | -5.36 (-7.98, -2.75) | **<.001** | -0.09 (-2.93, 2.75) | .951 | Not reported |  |
| Group A-R-E * Time | 8.07 (4.03, 12.11) | **<.001** | 2.34 (-1.91, 6.58) | .281 |  |  |
| Group R-A-E * Time | 5.38 (1.78, 8.98) | **.003** | -0.99 (-4.88, 2.91) | .620 |  |  |
| **30-sec arm curl test** |  |  |  |  |  |  |
| Time effect | -0.2 (-1.27, 0.88) | .721 | -0.35 (-1.51, 0.82) | .559 | 0.39 (-0.78, 1.55) | .514 |
| Group A-R-E * Time | 0.5 (-1.16, 2.16) | .553 | 2.17 (0.43, 3.91) | **.014** | 2.41 (0.49, 4.33) | **.014** |
| Group R-A-E * Time | 0.75 (-0.74, 2.23) | .325 | 1.82 (0.21, 3.44) | **.027** | 0.48 (-1.13, 2.09) | .560 |
| **2-minute step test** |  |  |  |  |  |  |
| Time effect | 0.3 (-7.52, 8.13) | .939 | 13.36 (4.9, 21.83) | **.002** | 5.36 (-3.12, 13.83) | .215 |
| Group A-R-E * Time | 8.68 (-3.37, 20.73) | .158 | 4.92 (-7.72, 17.56) | .446 | -5.86 (-19.83, 8.11) | .411 |
| Group R-A-E * Time | 2.52 (-8.26, 13.3) | .647 | -7.56 (-19.17, 4.05) | .202 | -6.13 (-17.79, 5.53) | .303 |
| **IADL** |  |  |  |  |  |  |
| Time effect | 0.31 (-0.47, 1.08) | .434 | -0.23 (-1.07, 0.61) | .591 | 0.18 (-0.66, 1.02) | .671 |
| Group A-R-E * Time | 0.43 (-0.78, 1.63) | .487 | 0.34 (-0.96, 1.61) | .604 | -0.79 (-2.20, 0.61) | .270 |
| Group R-A-E * Time | -0.33 (-1.40, 0.75) | .549 | -0.36 (-1.51, 0.79) | .543 | -1.17 (-2.33, -0.01) | **.048** |
| **PASE** |  |  |  |  |  |  |
| Time effect | 9.07 (-3.15, 21.30) | .146 | 2.88 (-10.44, 16.21) | .672 | 14.6 (1.39, 27.81) | **.030** |
| Group A-R-E * Time | -1.61 (-20.54, 17.32) | .868 | 0.57 (-19.32, 20.45) | .955 | -1.53 (-23.39, 20.33) | .891 |
| Group R-A-E * Time | 0 (-17.02,17.01) | 1.000 | 4.41 (-13.73, 22.56) | .633 | -2.57 (-20.75, 15.62) | .782 |
| **LSNS** |  |  |  |  |  |  |
| Time effect | 4.42 (1.94, 6.89) | **<.001** | 4.78 (2.10, 7.45) | **<.001** | 3.47 (0.79, 6.14) | **.011** |
| Group A-R-E * Time | -3.3 (-7.13, 0.53) | .091 | -2.89 (-6.92, 1.15) | .161 | -2.95 (-7.41, 1.51) | .194 |
| Group R-A-E * Time | -2.18 (-5.60, 1.25) | .213 | -2.86 (-6.53, 0.81) | .126 | -1.86 (-5.55, 1.83) | .323 |
| **PSQI** |  |  |  |  |  |  |
| Time effect | -0.73 (-1.70, 0.25) | .145 | -1.17 (-2.24, -0.09) | **.033** | -0.31 (-1.37, 0.75) | .564 |
| Group A-R-E * Time | 0.74 (-0.78, 2.27) | .339 | 1.84 (0.22, 3.46) | **.026** | -0.28 (-2.06, 1.51) | .761 |
| Group R-A-E * Time | 0.83 (-0.53, 2.19) | .234 | 0.87 (-0.60, 2.33) | .248 | 1.59 (0.12, 3.06) | **.034** |
| **PHQ-9** |  |  |  |  |  |  |
| Time effect | -1.55 (-3.08, -0.03) | **.046** | -0.26 (-1.93, 1.41) | .760 | -1.91 (-3.57, -0.26) | **.024** |
| Group A-R-E * Time | -0.27 (-2.63, 2.10) | .826 | 0.54 (-1.97, 3.04) | .675 | 1.33 (-1.43, 4.09) | .344 |
| Group R-A-E * Time | 0.83 (-1.28, 2.95) | .440 | 0.46 (-1.82, 2.74) | .693 | 2.5 (0.22, 4.79) | **.032** |

*Note.* A = Aerobic training. R = Resistance training. E = Lifestyle education.

IADL = Lawton’s Instrumental Activities of Daily Living Scale; LSNS = Lubben Social Network Scale; PASE = Physical Activity Scale for the Elderly; PHQ-9 = Patient Health Questionnaire; PSQI = Pittsburgh Sleep Quality Index; SPPB = Short Physical Performance Battery; WHOQoL-OLD = Cantonese version of the World Health Organization Quality of Life
