# Supplementary material for: Frailty, Fitness, and Quality of Life Outcomes of a Healthy and Productive Aging Program (GrandMove) for Older Adults With Frailty or Prefrailty: Cluster Randomized Controlled Trial
Source: JMIR Aging. 2025 May 14;8:e65636. doi: 10.2196/65636 (PMC12094531; doi:10.2196/65636)
Supplement: Multimedia Appendix 9 [file aging-v8-e65636-s009.docx]

**Multimedia Appendix 9.** Summary of time and group × time interaction effects on primary and secondary outcomes (female participants only)

|  | **Baseline to 6 months** | | **Baseline to 12 months** | | **Baseline to 18 months** | |
| --- | --- | --- | --- | --- | --- | --- |
|  | **Coefficient (95% or 9(% CI)** | **P-value** | **Coefficient (95% or 9(% CI)** | **P-value** | **Coefficient (95% or 9(% CI)** | **P-value** |
| **5-item FRAIL scale** |  |  |  |  |  |  |
| Time effect | -0.81 (-1.13, -0.50) | <.001 | -1.08 (-1.4, -0.75) | <.001 | -1.03 (-1.36, -0.70) | <.001 |
| Group A-R-E * Time | 0.11 (-0.36, 0.58) | .540 | 0.03 (-0.45, 0.51) | .866 | 0.04 (-0.47, 0.55) | .829 |
| Group R-A-E * Time | -0.09 (-0.54, 0.35) | .593 | 0.15 (-0.32, 0.61) | .420 | 0.05 (-0.43, 0.53) | .799 |
| **SPPB** |  |  |  |  |  |  |
| Time effect | -0.06 (-0.61, 0.50) | .794 | -0.62 (-1.19, -0.04) | .006 | -0.66 (-1.23, -0.08) | .003 |
| Group A-R-E * Time | 0.61 (-0.21, 1.44) | .056 | 0.83 (-0.03, 1.69) | .013 | 0.79 (-0.11, 1.69) | .023 |
| Group R-A-E * Time | 0.21 (-0.58, 1.00) | .495 | 0.39 (-0.43, 1.22) | .220 | 0.17 (-0.67, 1.02) | .596 |
| **WHOQOL-OLD** |  |  |  |  |  |  |
| Time effect | 0.12 (-3.04, 3.29) | .919 | 1.21 (-2.07, 4.50) | .342 | 0.42 (-2.89, 3.73) | .744 |
| Group A-R-E * Time | 2.67 (-2.03, 7.36) | .143 | -0.48 (-5.33, 4.37) | .799 | 1.55 (-3.58, 6.67) | .436 |
| Group R-A-E * Time | 4.64 (0.15, 9.13) | .008 | 3.84 (-0.87, 8.55) | .036 | 3.11 (-1.73, 7.94) | .098 |
| **Grip strength (left hand)** |  |  |  |  |  |  |
| Time effect | -3.36 (-4.98, -1.74) | <.001 | -0.79 (-2.48, 0.90) | .360 | Not reported |  |
| Group A-R-E * Time | 4.17 (1.77, 6.57) | .001 | 1.23 (-1.28, 3.75) | .336 |  |  |
| Group R-A-E * Time | 4.19 (1.89, 6.49) | <.001 | 1.25 (-1.17, 3.67) | .310 |  |  |
| **Grip strength (right hand)** |  |  |  |  |  |  |
| Time effect | -3.62 (-5.22, -2.02) | <.001 | -1.42 (-3.09, 0.24) | .093 | Not reported |  |
| Group A-R-E * Time | 4.69 (2.31, 7.07) | <.001 | 1.82 (-0.67, 4.30) | .152 |  |  |
| Group R-A-E * Time | 3.94 (1.66, 6.22) | .001 | 0.88 (-1.51, 3.27) | .472 |  |  |
| **30-sec arm curl** |  |  |  |  |  |  |
| Time effect | 0.16 (-0.62, 0.94) | .685 | 0.22 (-0.58, 1.03) | .588 | 0.33 (-0.48, 1.14) | .419 |
| Group A-R-E * Time | 1.07 (-0.08, 2.22) | .068 | 0.83 (-0.37, 2.03) | .174 | 0.16 (-1.10, 1.42) | .802 |
| Group R-A-E * Time | 0.66 (-0.29, 2.04) | .237 | 0.87 (-0.29, 2.04) | .141 | 0.54 (-0.64, 1.73) | .368 |
| **2-minute step test** |  |  |  |  |  |  |
| Time effect | 2.20 (-3.01, 7.42) | .408 | 7.52 (2.12, 12.93) | .006 | 6.87 (1.40, 12.34) | .014 |
| Group A-R-E * Time | 7.82 (0.07, 15.58) | .048 | 5.61 (-2.47, 13.69) | .174 | 4.16 (-4.27, 12.60) | .333 |
| Group R-A-E * Time | 5.79 (-1.61, 13.19) | .125 | 0.58 (-7.16, 8.33) | .883 | 0.64 (-7.34, 8.62) | .875 |
| **IADL** |  |  |  |  |  |  |
| Time effect | 0.41 (-0.08, 0.90) | .098 | 0.78 (0.27, 1.29) | .003 | 0.65 (0.14, 1.16) | .013 |
| Group A-R-E * Time | -0.67 (-0.79, 0.66) | .857 | -0.63 (-1.39, 0.12) | .101 | -0.60 (-1.39, 0.20) | .141 |
| Group R-A-E * Time | -0.04 (-0.73, 0.66) | .919 | -0.74 (-1.47, -.01) | .047 | -0.66 (-1.41, 0.09) | .086 |
| **PASE** |  |  |  |  |  |  |
| Time effect | 4.43 (-4.65, 13.50) | .339 | 7.11 (-2.30, 16.53) | .139 | 9.61 (0.19, 19.02) | .045 |
| Group A-R-E * Time | 10.28 (-3.14, 23.70) | .133 | -0.54 (-14.39, 13.31) | .939 | 1.70 (-12.82, 16.21) | .819 |
| Group R-A-E * Time | -2.33 (-15.23, 10.56) | .723 | -3.09 (-16.53, 10.35) | .653 | -1.35 (-15.07, 12.37) | .847 |
| **LSNS** |  |  |  |  |  |  |
| Time effect | 0.92 (-0.79, 2.62) | .292 | 2.87 (1.10, 4.63) | .001 | 1.12 (-0.66, 2.90) | .219 |
| Group A-R-E * Time | -1.24 (-3.76, 1.29) | .338 | -2.04 (-4.65, 0.58) | .127 | -0.58 (-3.34, 2.19) | .683 |
| Group R-A-E * Time | 0.20 (-0.21, 2.61) | .870 | -0.92 (-3.45, 1.62) | .479 | 2.22 (-0.38, 4.83) | .094 |
| **PSQI** |  |  |  |  |  |  |
| Time effect | -0.59 (-1.27, 0.08) | .086 | -0.88 (-1.59. -0.17) | .015 | 0.11 (-0.60, 0.82) | .763 |
| Group A-R-E * Time | -0.22 (-1.24, 0.80) | .673 | 0.51 (-0.55, 1.57) | .345 | -0.89 (-2.01, 0.22) | .117 |
| Group R-A-E * Time | 0.23 (-0.75, 1.20) | .649 | 0.62 (-0.41, 1.64) | .237 | 0.36 (-0.69, 1.41) | .499 |
| **PHQ-9** |  |  |  |  |  |  |
| Time effect | -1.17 (-2.18, 0.15) | .025 | -1.09 (-2.15, -0.04) | .043 | -1.80 (-2.86, -0.74) | .001 |
| Group A-R-E * Time | -0.06 (-1.56, 1.45) | .941 | -0.24 (-1.80, 1.32) | .762 | 0.17 (-1.47, 1.82) | .835 |
| Group R-A-E * Time | 0.71 (-0.73, 2.15) | .334 | 0.90 (-0.61, 2.42) | .243 | 1.66 (0.10, 3.21) | .037 |

*Note.* A = Aerobic training. R = Resistance training. E = Lifestyle education.

IADL = Lawton’s Instrumental Activities of Daily Living Scale; LSNS = Lubben Social Network Scale; PASE = Physical Activity Scale for the Elderly; PHQ-9 = Patient Health Questionnaire; PSQI = Pittsburgh Sleep Quality Index; SPPB = Short Physical Performance Battery; WHOQoL-OLD = Cantonese version of the World Health Organization Quality of Life - Older Adults Module.
