# Supplementary material for: Frailty, Fitness, and Quality of Life Outcomes of a Healthy and Productive Aging Program (GrandMove) for Older Adults With Frailty or Prefrailty: Cluster Randomized Controlled Trial
Source: JMIR Aging. 2025 May 14;8:e65636. doi: 10.2196/65636 (PMC12094531; doi:10.2196/65636)
Supplement: Multimedia Appendix 10 [file aging-v8-e65636-s010.docx]

**Multimedia Appendix 10.** Summary of time and group × time interaction effects on primary and secondary outcomes (male participants only)

|  | **Baseline to 6 months** | | **Baseline to 12 months** | | **Baseline to 18 months** | |
| --- | --- | --- | --- | --- | --- | --- |
|  | **Coefficient (95% or 9(% CI)** | **P-value** | **Coefficient (95% or 9(% CI)** | **P-value** | **Coefficient (95% or 9(% CI)** | **P-value** |
| **5-item FRAIL scale** |  |  |  |  |  |  |
| Time effect | -0.62 (-1.24, 0.01) | .011 | -1.02 (-1.66, -0.38) | <.001 | -1.27 (-2.02, -0.52) | <.001 |
| Group A-R-E * Time | -0.54 (-1.41, 0.33) | .111 | 0.18 (-0.72, 1.09) | .601 | 0.59 (-0.43, 1.60) | .135 |
| Group R-A-E * Time | -0.29 (-1.15, 0.56) | .380 | 0.05 (-0.84. 0.94) | .881 | 0.59 (-0.39, 1.57) | .119 |
| **SPPB** |  |  |  |  |  |  |
| Time effect | -0.68 (-1.77, 0.40) | .105 | -0.47 (-1.63, 0.70) | .301 | -0.37 (-1.71, 0.97) | .476 |
| Group A-R-E * Time | 0.43 (-1.09, 1.95) | .464 | 0.27 (-1.34, 1.89) | .662 | -0.42 (-2.24, 1.40) | .555 |
| Group R-A-E * Time | 0.92 (-0.56, 2.40) | .109 | 0.19 (-1.40, 1.78) | .761 | -0.55 (-2.28, 1.19) | .415 |
| **WHOQOL-OLD** |  |  |  |  |  |  |
| Time effect | 0.07 (-6.18, 6.31) | .977 | 1.01 (-5.42, 7.44) | .686 | -1.14 (-8.67, 6.39) | .697 |
| Group A-R-E * Time | 7.38 (-1.38, 16.15) | .030 | 2.59 (-6.50, 11.68) | .463 | 4.17 (-6.02, 14.36) | .292 |
| Group R-A-E * Time | 8.16 (-0.43, 16.75) | .014 | 7.53 (-1.14, 16.48) | .030 | 9.26 (-0.63, 19.15) | .016 |
| **Grip strength (left hand)** |  |  |  |  |  |  |
| Time effect | -0.57 (-4.33, 3.20) | .766 | 2.27 (-1.78, 6.32) | .271 | Not reported |  |
| Group A-R-E * Time | 2.11 (-3.27, 7.49) | .442 | -0.62 (-6.36, 5.12) | .833 |  |  |
| Group R-A-E * Time | -2.19 (-7.37, 2.98) | .407 | -4.50 (-10.04, 1.04) | .111 |  |  |
| **Grip strength (right hand)** |  |  |  |  |  |  |
| Time effect | -1.13 (-4.87, 2.60) | .551 | -0.17 (-4.18, 3.84) | .935 | Not reported |  |
| Group A-R-E * Time | 2.56 (-2.71, 7.84) | .340 | 1.00 (-4.62, 6.61) | .728 |  |  |
| Group R-A-E * Time | -1.85 (-6.94, 3.24) | .476 | -3.53 (-9.02, 1.95) | .207 |  |  |
| **30-sec arm curl** |  |  |  |  |  |  |
| Time effect | -0.96 (-2.58, 0.66) | .247 | -0.12 (-1.86, 1.62) | .893 | -0.93 (-2.93, 1.07) | .363 |
| Group A-R-E * Time | 3.08 (0.80, 5.37) | .008 | 3.55 (1.13, 5.96) | .004 | 2.37 (-0.35, 5.08) | .088 |
| Group R-A-E * Time | 3.10 (0.88, 5.31) | .006 | 1.86 (-0.52, 4.24) | .125 | 1.62 (-0.98, 4.23) | .222 |
| **2-minute step test** |  |  |  |  |  |  |
| Time effect | 3.58 (-7.13, 14.28) | .513 | 9.29 (-2.19, 20.78) | .113 | 9.03 (-4.17, 22.24) | .180 |
| Group A-R-E * Time | 15.05 (-0.05, 30.15) | .051 | 11.62 (-4.60, 27.83) | .160 | -2.05 (-20.01, 15.91) | .823 |
| Group R-A-E * Time | -0.36 (-14.97, 14.26) | .962 | -2.09 (-17.79, 13.62) | .795 | -11.27 (-28.38, 5.84) | .197 |
| **IADL** |  |  |  |  |  |  |
| Time effect | 0.74 (-0.33, 1.81) | .177 | 0.30 (-0.80, 1.41) | .593 | 1.18 (-1.12, 2.48) | .075 |
| Group A-R-E * Time | -0.08 (-1.58, 1.42) | .914 | -0.61 (-2.17, 0.95) | .442 | -1.29 (-3.05, 0.46) | .148 |
| Group R-A-E * Time | -0.14 (-1.61, 1.33) | .854 | -0.67 (-2.20, 0.87) | .396 | -2.56 (-4.25, -0.87) | .003 |
| **PASE** |  |  |  |  |  |  |
| Time effect | -12.54 (-31.77, 6.69) | .201 | -14.44 (-33.98, 5.11) | .148 | 31.42 (8.71, 54.13) | .007 |
| Group A-R-E * Time | 9.38 (-17.42, 36.18) | .493 | 15.17 (-12.42, 42.75) | .281 | -27.72 (-58.53, 3.10) | .078 |
| Group R-A-E * Time | 37.17 (11.05, 63.28) | .005 | 27.24 (0.22, 54.26) | .048 | -11.25 (-40.82, 18.32) | .456 |
| **LSNS** |  |  |  |  |  |  |
| Time effect | 1.04 (-2.65, 4.72) | .581 | 1.96 (-1.84, 5.75) | .312 | 4.42 (-0.02, 8.87) | .051 |
| Group A-R-E * Time | 0.73 (-4.44, 5.89) | .783 | -0.27 (5.63, 5.09) | .922 | -2.03 (-8.05, 3.98) | .508 |
| Group R-A-E * Time | 1.11 (-3.95, 6.18) | .666 | 0.17 (-5.11, 5.45) | .951 | -4.37 (-10.17, 1.43) | .140 |
| **PSQI** |  |  |  |  |  |  |
| Time effect | -1.27 (-2.56, 0.03) | .055 | -0.95 (-2.28, 0.38) | .163 | 0.34 (-1.22, 1.91) | .666 |
| Group A-R-E * Time | 1.59 (-0.24, 3.41) | .088 | 1.75 (-0.15, 3.64) | .070 | 0.39 (-1.73, 2.51) | .715 |
| Group R-A-E * Time | 0.24 (-1.58, 2.06) | .799 | 0.62 (-1.28, 2.53) | .520 | 0.40 (-1.69, 2.48) | .709 |
| **PHQ-9** |  |  |  |  |  |  |
| Time effect | -0.71 (-2.26, 0.85) | .373 | -0.35 (-1.95, 1.25) | .670 | 0.01 (-1.87, 1.89) | .990 |
| Group A-R-E * Time | -0.16 (-2.34, 2.02) | .884 | 0.79 (-1.47, 3.05) | .492 | 0.95 (-1.59, 3.49) | .463 |
| Group R-A-E * Time | -0.45 (-2.58, 1.69) | .682 | -0.49 (-2.73, 1.76) | .669 | 0.10 (-2.37, 2.56) | .940 |

*Note.* A = Aerobic training. R = Resistance training. E = Lifestyle education.

IADL = Lawton’s Instrumental Activities of Daily Living Scale; LSNS = Lubben Social Network Scale; PASE = Physical Activity Scale for the Elderly; PHQ-9 = Patient Health Questionnaire; PSQI = Pittsburgh Sleep Quality Index; SPPB = Short Physical Performance Battery; WHOQoL-OLD = Cantonese version of the World Health Organization Quality of Life - Older Adults Module
