# Supplementary material for: Frailty, Fitness, and Quality of Life Outcomes of a Healthy and Productive Aging Program (GrandMove) for Older Adults With Frailty or Prefrailty: Cluster Randomized Controlled Trial
Source: JMIR Aging. 2025 May 14;8:e65636. doi: 10.2196/65636 (PMC12094531; doi:10.2196/65636)
Supplement: Multimedia Appendix 11 [file aging-v8-e65636-s011.docx]

**Multimedia Appendix 11.** Comparison of outcomes between Group R-A-E and Group A-R-E (reference) at 12-month follow-up

|  | Group R-A-E vs Group A-R-E (reference) at 12 months | | | | | |
| --- | --- | --- | --- | --- | --- | --- |
|  | Prefrail and frail participants | | Prefrail participants | | Frail participants | |
|  | Coefficient (95% CI) | P-value | Coefficient (95% CI) | P-value | Coefficient (95% CI) | P-value |
| FRAIL score | 0.09 (-0.35, 0.52) | .61 | 0.36 (-0.13, 0.86) | .86 | -0.35 (-1.04, 0.34) | .19 |
| SPPB | -0.23 (-0.92, 0.46) | .39 | -0.34 (-1.27, 0.59) | .35 | -0.35 (-1.66, 0.96) | .49 |
| WHOQoL-OLD | 4.50 (0.12, 8.88) | .008 | 7.11 (2.02, 12.20) | <.001 | 0.46 (-7.27, 8.18) | .88 |
| Grip strength (left) | -0.75 (-3.11, 1.60) | .53 | 0.31 (-2.34, 2.96) | .82 | -3.44 (-7.77, 0.88) | .12 |
| Grip strength (right) | -1.56 (-3.90, 0.78) | .19 | -1.09 (-3.80, 1.62) | .43 | -0.32 (-7.45, 0.80) | .11 |
| Arm curl | -0.43 (-1.52, 0.66) | .44 | -0.40 (-1.78, 0.98) | .57 | -0.35 (-2.06, 1.36) | .69 |
| 2-minute step test | -5.66 (-12.76, 1.45) | .12 | -2.94 (-11.81, 5.92) | .52 | -12.48 (-24.78, -0.18) | .047 |
| IADL | -0.33 (-0.99, 0.34) | .34 | 0.38 (-0.43, 1.19) | .36 | -0.69 (-1.93, 0.54) | .27 |
| PASE | 0.51 (-12.4, 13.4) | .94 | -0.63 (-17.17, 15.91) | .94 | 3.85 (-15.38, 23.08) | .70 |
| LSNS | 0.54 (-1.86, 2.94) | .66 | 1.49 (-1.49, 4.46) | .33 | 0.03 (-3.90, 3.95) | .99 |
| PSQI | -0.22 (-1.17, 0.73) | .65 | 0.16 (-1.01, 1.33) | .79 | -0.98 (-2.55, 0.60) | .23 |
| PHQ-9 | 1.02 (-0.26, 2.30) | .12 | 0.70 (-0.86, 2.27) | .38 | -0.08 (-2.50, 2.35) | .9s5 |

*Note.* A = Aerobic training. R = Resistance training. E = Lifestyle education.
IADL = Lawton’s Instrumental Activities of Daily Living Scale; LSNS = Lubben Social Network Scale; PASE = Physical Activity Scale for the Elderly; PHQ-9 = Patient Health Questionnaire; PSQI = Pittsburgh Sleep Quality Index; SPPB = Short Physical Performance Battery; WHOQoL-OLD = Cantonese version of the World Health Organization Quality of Life - Older Adults Module.
